# Video-Assisted Thoracoscopic Surgery (VATS)-Aided Simultaneous Fixation of Scapular Body and Rib Fractures: A Case Report

**DOI:** 10.7759/cureus.81013

**Published:** 2025-03-22

**Authors:** Masahiro Matsumoto, Hyonmin Choe, Naomi Kobayashi, Ichiro Takeuchi, Yutaka Inaba

**Affiliations:** 1 Advanced Critical Care and Emergency Center, Yokohama City University Medical Center, Yokohama, JPN; 2 Orthopaedic Surgery, Yokohama City University School of Medicine, Yokohama, JPN; 3 Orthopaedic Surgery, Yokohama City Universty Medical Center, Yokohama, JPN

**Keywords:** modified judet approach, rib fracture, scapular body fracture, surgical stabilization of rib fractures(ssrf), video-assisted thoracoscopic surgery (vats)

## Abstract

Scapular fractures are rare injuries, whereas rib fractures are more common. Scapular fractures are frequently associated with rib fractures, which may lead to significant respiratory compromise. While surgical fixation has demonstrated benefits for both scapular and rib fractures, simultaneous fixation using a single surgical approach is not widely practiced. A 54-year-old male sustained a right scapular body fracture (AO 14B1) and multiple right rib fractures following a crush injury. Due to severe respiratory pain and prolonged mechanical ventilation, surgical fixation was performed on post-injury day three using a video-assisted thoracoscopic surgery (VATS)-aided modified Judet approach. The third to fifth rib fractures were stabilized with the Matrix RIB™ Fixation System (Johnson & Johnson, New Brunswick, NJ) and the scapular body fracture was fixed with locking compression plates. Postoperatively, the patient was extubated on day eight and discharged to rehabilitation on day 29. At the follow-up at one year and six months, complete bone union was confirmed with no residual functional impairment. The VATS-aided modified Judet approach enabled simultaneous fixation of scapular and rib fractures, potentially reducing surgical invasiveness, improving postoperative respiratory function, and expediting rehabilitation. This technique presents a viable option for managing complex thoracic fractures, warranting further investigation to evaluate its long-term efficacy.

## Introduction

Scapular fractures are exceedingly rare, accounting for approximately 0.4-1% of all fractures and 3-5% of upper limb fractures [1]. These fractures typically result from high-energy trauma, such as motor vehicle accidents and falls from height, and are often accompanied by concomitant injuries [2]. Among these, rib fractures are the most frequently associated injuries, occurring in 50-60% of scapular fracture cases [3]. Rib fractures can lead to severe chest pain, respiratory impairment, and increased risk of complications such as pneumonia and acute respiratory distress syndrome (ARDS) [4]. The presence of both scapular and rib fractures exacerbates these risks, often necessitating prolonged mechanical ventilation and delaying rehabilitation [5].

Traditionally, conservative treatment has been the standard approach for both scapular and rib fractures. However, recent studies have demonstrated that surgical fixation of rib fractures can improve respiratory function, reduce pain, shorten mechanical ventilation duration, and facilitate early mobilization [6-9]. Similarly, surgical fixation of scapular fractures has been reported to yield better functional outcomes in select cases, particularly when there is significant displacement or instability [10-13]. Despite these advancements, simultaneous surgical fixation of both scapular and rib fractures remains uncommon, primarily due to the complexity of achieving adequate exposure using a single approach [9].

In this report, we describe a novel approach utilizing a video-assisted thoracoscopic surgery (VATS)-aided modified Judet approach for the simultaneous fixation of a scapular body fracture and multiple rib fractures. This technique enabled effective stabilization of both fracture sites while minimizing surgical invasiveness. We discuss the surgical procedure, postoperative course, and potential advantages of this approach, emphasizing its role in optimizing respiratory recovery and functional outcomes in patients with combined scapular and rib fractures.

## Case presentation

**Figure 1:**
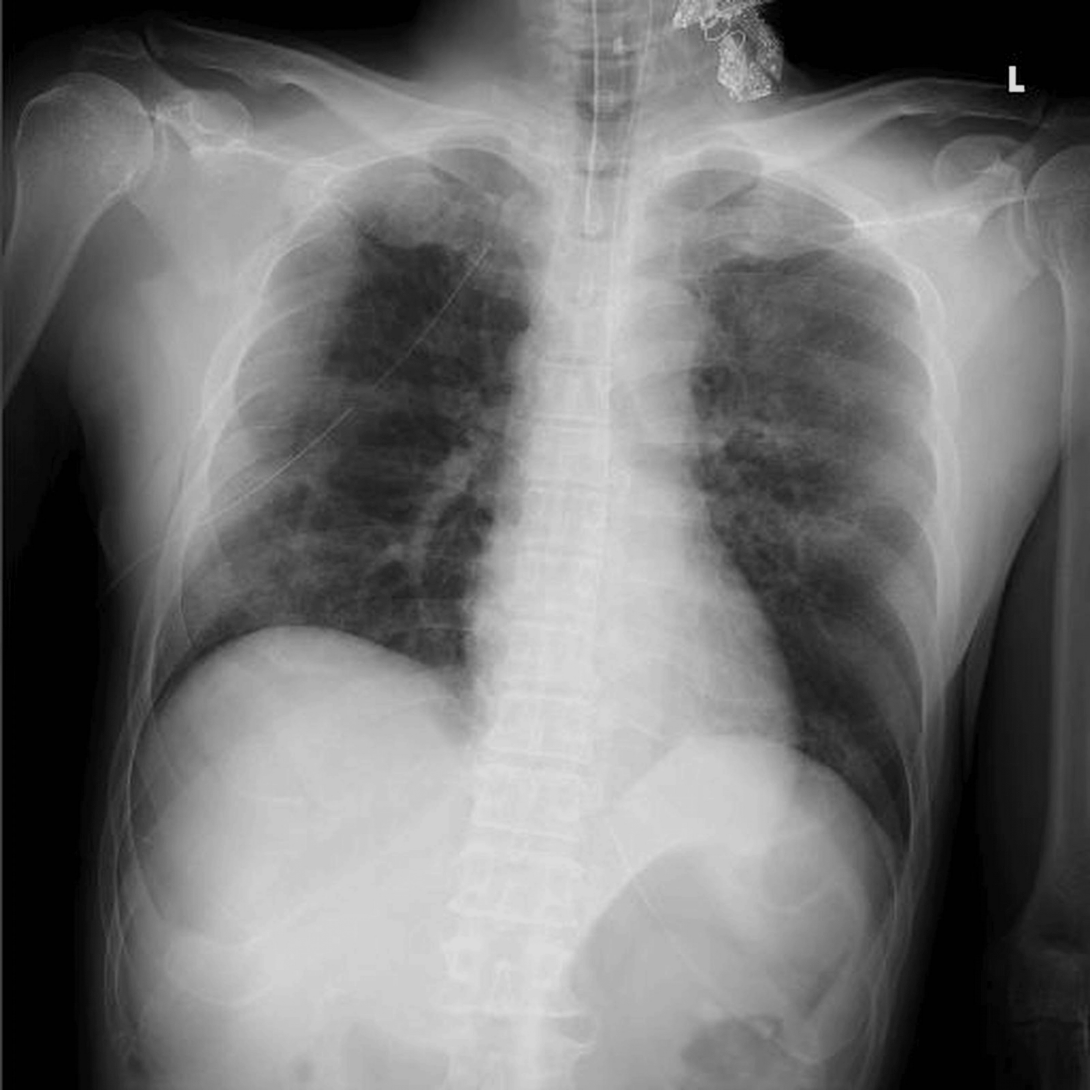
Chest X-ray image on admission The patient was intubated, and a chest drain was placed in the right pleural cavity

A 54-year-old male sustained crush injuries and was transported to our Advanced Critical Care and Emergency Center. A chest tube was placed in the emergency department, and the patient required tracheal intubation and mechanical ventilation (Figure 1).

**Figure 2:**
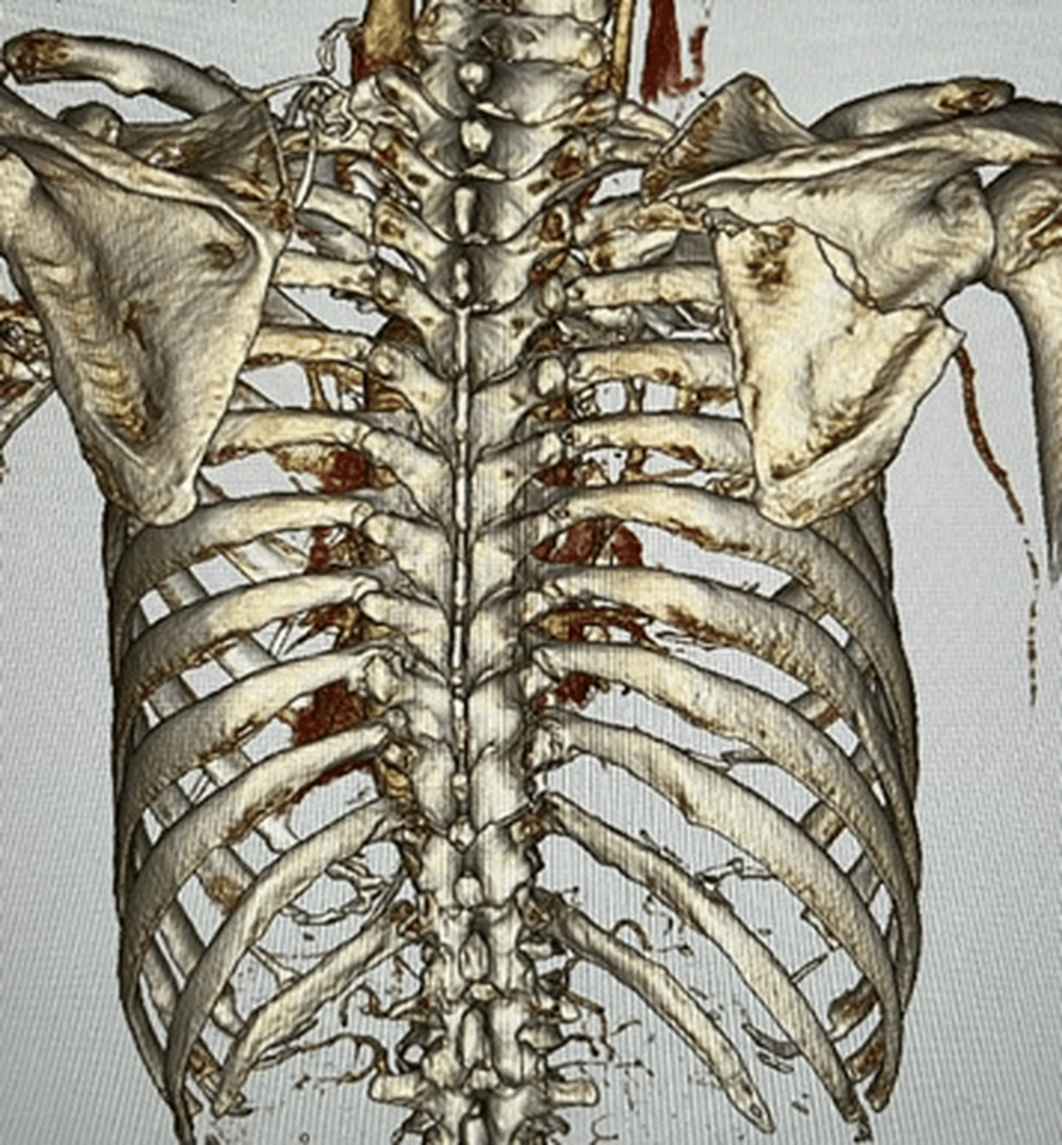
3D CT image on admission - 1 A fracture of the right scapular body was present, with multiple rib fractures accompanied by a flail segment just below it CT: computed tomography

**Figure 3:**
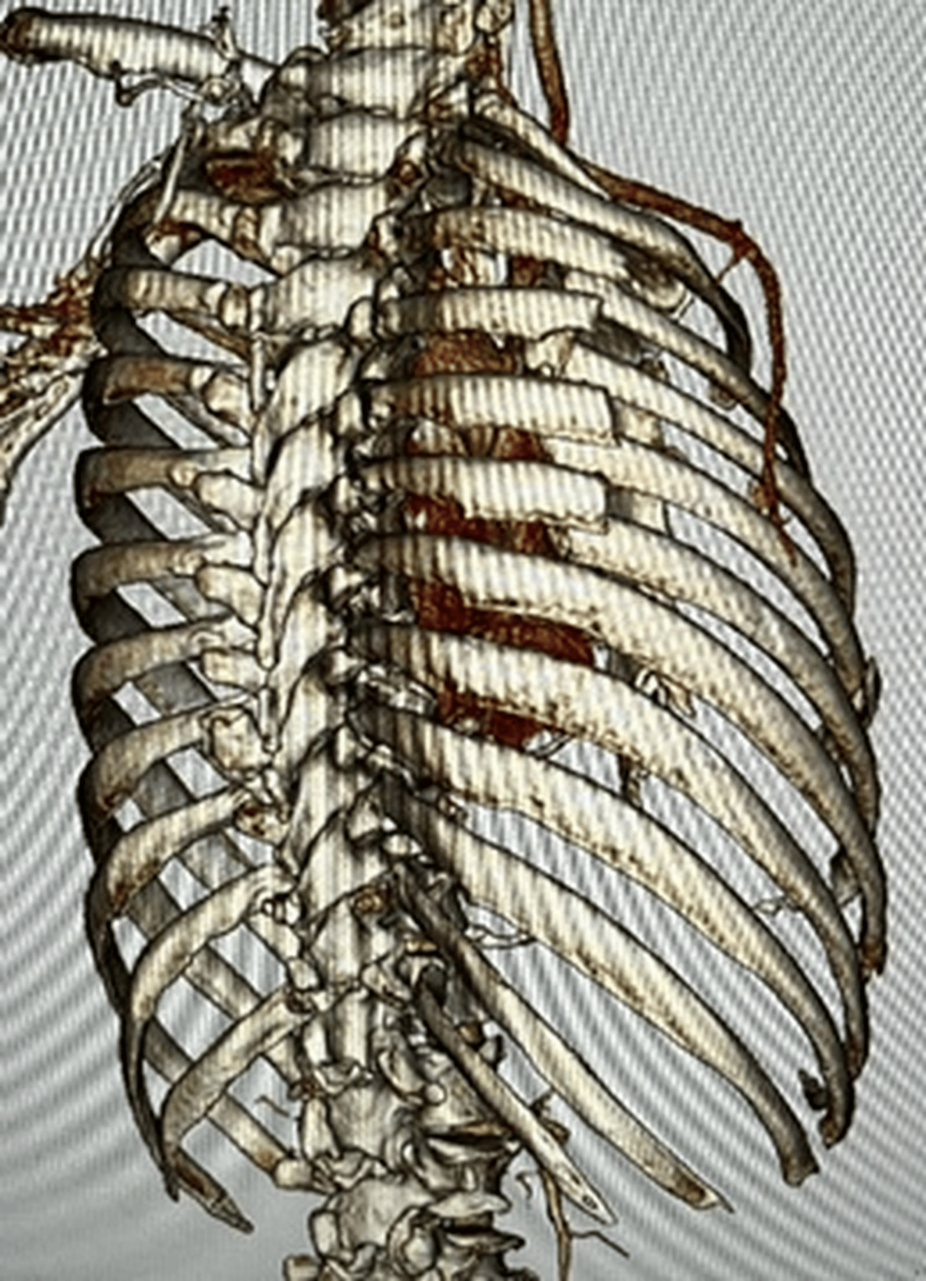
3D CT image on admission - 2 A fracture of the right scapular body was present, with multiple rib fractures accompanied by a flail segment just below it CT: computed tomography

A trauma pan-scan CT revealed the right multiple rib fractures, a right scapular body fracture (AO 14B1), a lumbar vertebral fracture, and a knee lateral collateral ligament (LCL) injury (Figures 2, 3).

The injury severity score (ISS) was 24, with a probability of survival (Ps) of 0.98. Due to persistent severe respiratory pain, early extubation was deemed difficult, and surgical intervention was performed on the third-day post-injury (64 hours after trauma).

Surgical procedure

**Figure 4:**
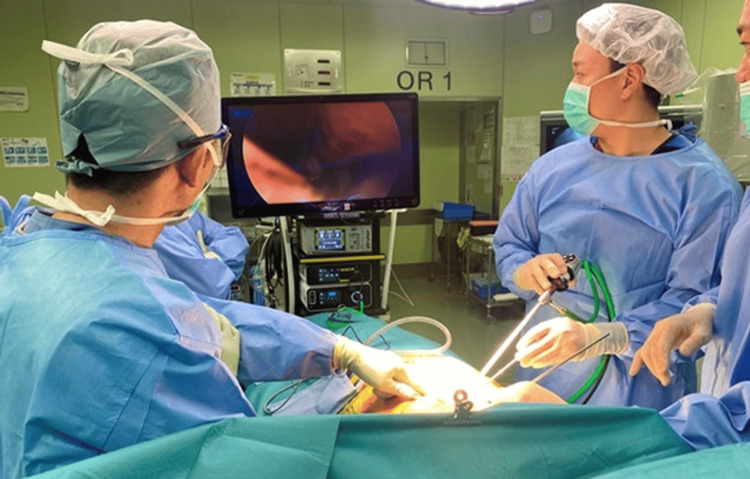
Surgery performed under VATS The rib fracture site is identified intrathoracically using VATS VATS: video-assisted thoracoscopic surgery

The patient was placed in a lateral decubitus position. First, VATS was introduced into the lateral thoracic region, 2-3 intercostal spaces away from the skin incision site, to observe the thoracic cavity and assess for lung injury. Subsequently, the rib fracture site was identified, confirmed through palpation from the body surface, and evaluated for instability (Figure 4).

**Figure 5:**
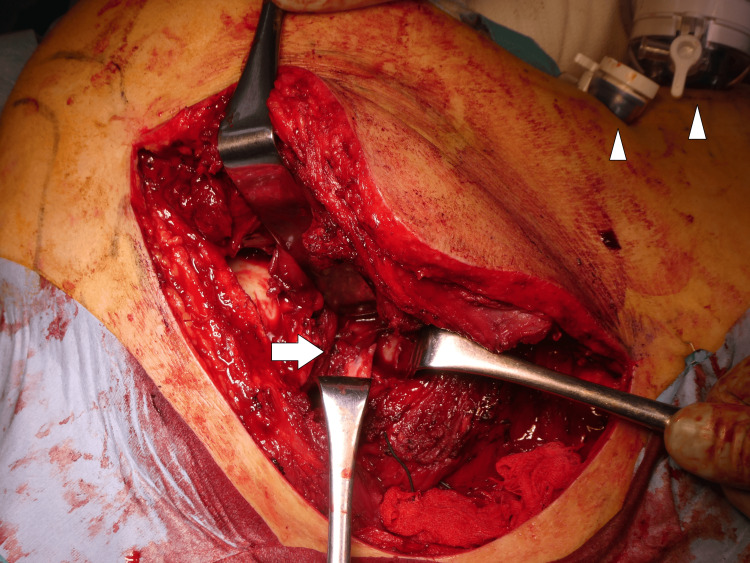
Intraoperative findings - 1 When the scapula was inverted at the site of the fracture, the rib fracture was identified (arrow). A VATS port is inserted in the lateral thoracic region (diagrams) VATS: video-assisted thoracoscopic surgery

**Figure 6:**
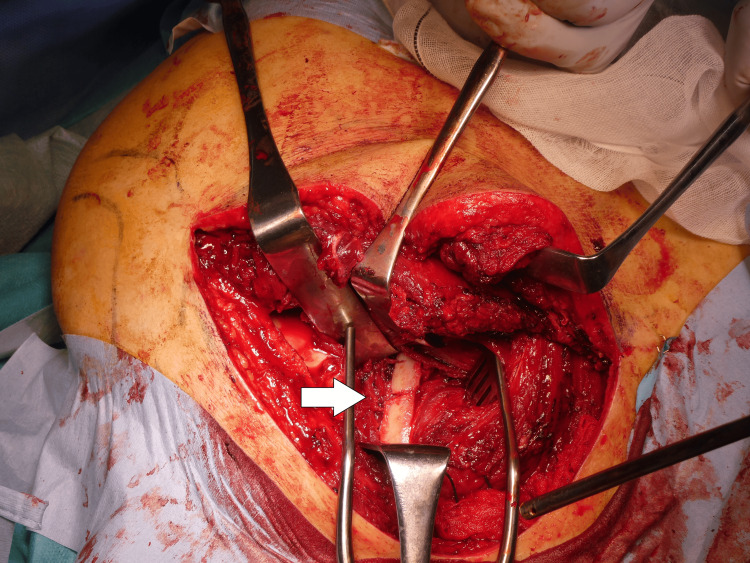
Intraoperative findings - 2 The rib fracture was reduced under direct visualization of the thoracic cavity using VATS (arrow) VATS: video-assisted thoracoscopic surgery

**Figure 7:**
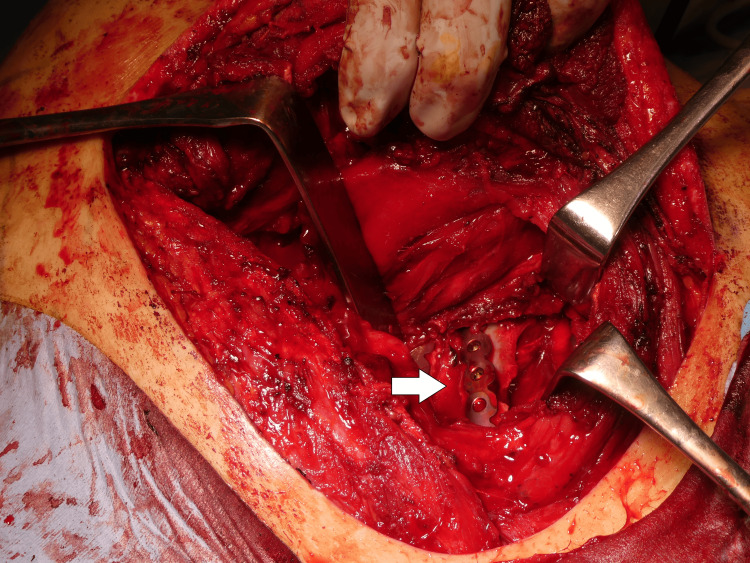
Intraoperative findings - 3 The rib fracture was stabilized using a rib-specific plate (arrow) VATS: video-assisted thoracoscopic surgery

**Figure 8:**
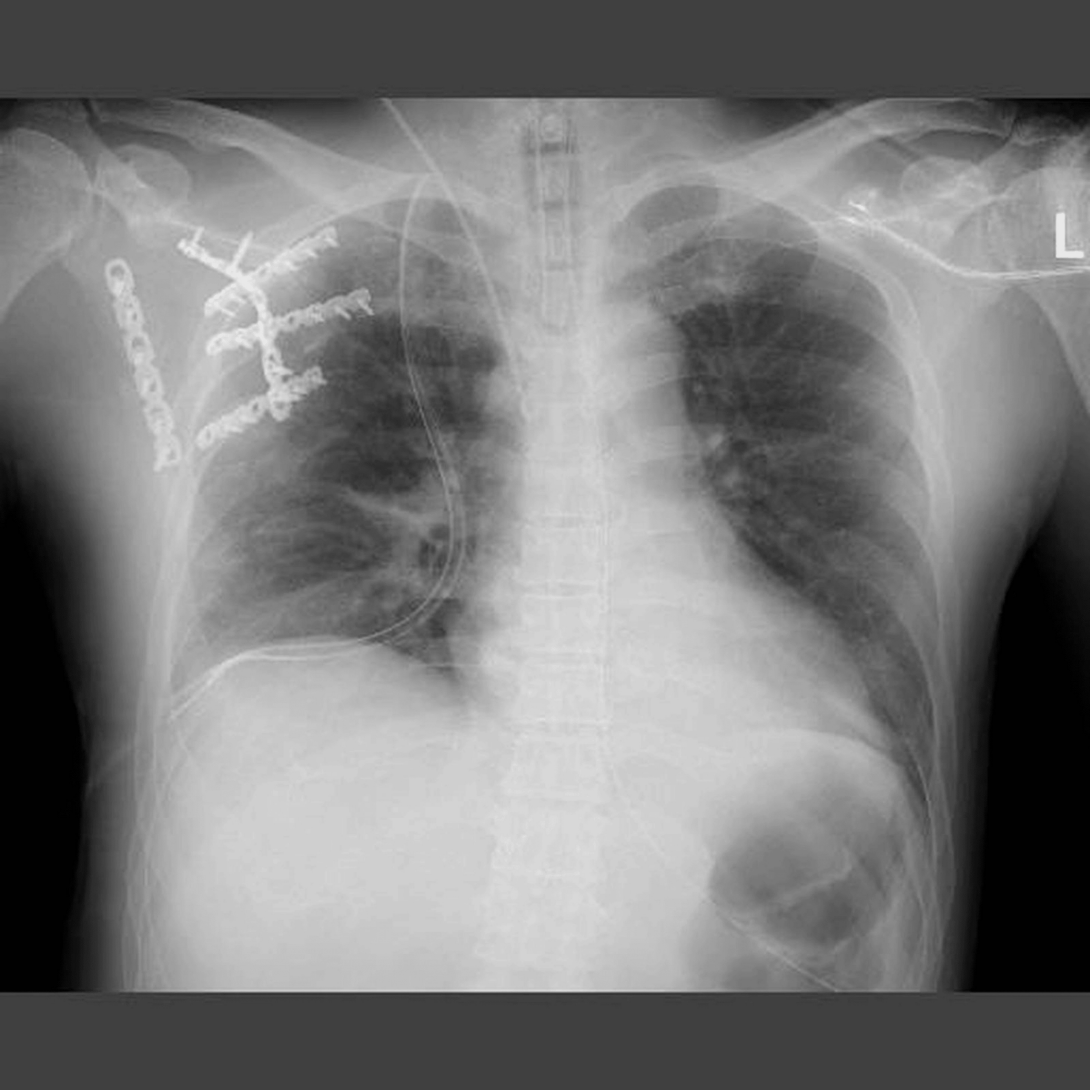
Postoperative chest and scapula X-ray images

The approach used was the modified Judet approach. A longitudinal skin incision was made, and the deltoid was partially detached from the scapular spine. The surgical field was expanded between the infraspinatus and teres minor muscles. The rhomboid major, attached to the distal scapular fragment, was partially dissected, and the scapular body was flipped caudally to expose the rib fractures. Under VATS guidance, the rib fractures were identified and reduced. The third to fifth ribs were fixed using the Matrix RIB™ Fixation System (Johnson & Johnson, New Brunswick, NJ). The parietal pleura was carefully repaired, and the medial and lateral borders of the scapula were plated using LCP® (Locking Compression Plate, Depuy Synthes, Raynham, MA). The medial border plate was bent along the scapular spine, while a straight plate was used for the lateral border. The wound was irrigated with normal saline, and the thoracic cavity was similarly irrigated. Before wound closure, the thoracic cavity was re-evaluated using VATS, and a thoracic drain was inserted through the VATS port. The pleura was repaired as much as possible, and the wound was closed by suturing the fascia and subcutaneous layers in a layered manner (Figures 5-8).

Postoperative course

**Figure 9:**
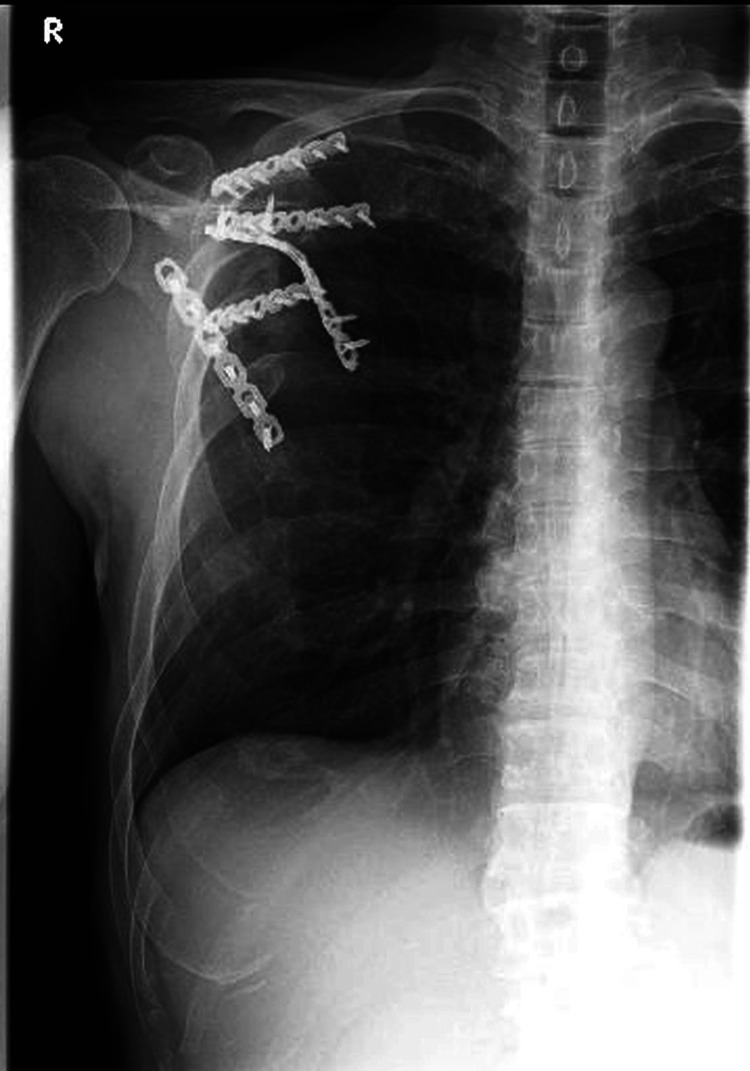
Chest and scapular X-rays at 1.5 years postoperatively The chest X-ray shows no abnormalities, and the rib fractures have achieved bone union. The scapular fracture has also healed, with no abnormalities observed in the implants

Chest wall stabilization was achieved postoperatively, and the patient was extubated and weaned off mechanical ventilation on postoperative day eight. Passive range-of-motion exercises, including shoulder elevation and abduction up to 90°, were initiated within the first two postoperative weeks. Active rehabilitation was then permitted without restriction. The patient was transferred to a rehabilitation hospital on day 29. At one year and six months postoperatively, there was no residual shoulder dysfunction, and radiographic evaluations confirmed the bony union of the scapula and ribs​ (Figure 9).

## Discussion

Scapular fractures are relatively uncommon, accounting for 0.4-1% of all fractures and 3-5% of upper limb fractures [1]. These fractures typically result from high-energy trauma, such as motor vehicle accidents, falls from height, or direct impact injuries [2]. Due to the strong surrounding musculature and the thoracic cage, scapular fractures rarely occur in isolation, with rib fractures being the most frequently associated injury, occurring in up to 60% of cases [3]. When combined, scapular and rib fractures significantly impair chest wall stability and respiratory function, increasing the risk of atelectasis, pneumonia, and ARDS [4]. Given these risks, treatment strategies must be carefully considered to optimize both orthopedic and pulmonary outcomes.

Historically, conservative management has been the preferred approach for both scapular and rib fractures, particularly in cases without significant displacement or mechanical instability [5]. However, recent studies have increasingly supported surgical fixation, particularly for severely displaced scapular fractures and multiple rib fractures with flail chest or respiratory compromise [6-7,10-11]. Surgical rib fixation has been shown to improve respiratory mechanics, reduce pain, shorten the duration of mechanical ventilation, and facilitate early mobilization [8-9,14-15]. Similarly, surgical stabilization of scapular fractures has demonstrated superior functional outcomes in cases involving glenoid involvement, significant displacement, or scapulothoracic dissociation [10-13]. Despite these benefits, simultaneous fixation of scapular and rib fractures remains uncommon, as it presents unique challenges in terms of surgical exposure, approach selection, and minimizing invasiveness.

In this case, a VATS-aided modified Judet approach was employed to enable simultaneous fixation of scapular and rib fractures. There have been several reports on the advantages of VATS-aided rib fixation, and the WSES and CWIS positioning paper also states that its consideration may be appropriate depending on the case [16-18]. This approach provides several advantages over conventional methods:

Advantages over conventional methods

Optimized Surgical Exposure

The modified Judet approach allowed for adequate visualization of the scapular body and lateral thoracic wall, while VATS facilitated direct inspection of the pleural cavity, reducing the risk of pleural injury and intraoperative complications.

Enhanced Chest Wall Stability

Concurrent fixation of both scapular and rib fractures restored thoracic integrity, leading to improved respiratory mechanics and earlier weaning from mechanical ventilation.

Minimized Surgical Invasiveness

The combination of VATS and a single-incision approach reduced surgical trauma and the need for multiple procedures, which may otherwise increase postoperative morbidity and delay rehabilitation.

Facilitated Early Mobilization

By stabilizing both the scapula and ribs, this approach enabled the early initiation of physical therapy, promoting better functional recovery and shoulder mobility.

Limitations

While the VATS-aided modified Judet approach demonstrated several advantages in this case, certain limitations must be acknowledged. Firstly, this report involves a single case, and further prospective studies are needed to evaluate its long-term efficacy and reproducibility in a larger cohort. Second, not all scapular and rib fractures require surgical intervention, and careful patient selection remains essential to maximize the benefits of surgical fixation. Additionally, VATS-aided techniques require specialized training and equipment, which may limit their widespread adoption in centers without thoracic surgery expertise.

## Conclusions

The VATS-aided modified Judet approach represents a viable and effective surgical technique for the simultaneous fixation of scapular and rib fractures. By enhancing surgical exposure, stabilizing the chest wall, and facilitating early rehabilitation, this approach has the potential to improve both orthopedic and respiratory outcomes. Further research is warranted to establish its long-term clinical benefits and broader applicability in complex thoracic trauma cases.
